# Higher TIGIT+ γδ T_CM_ cells may predict poor prognosis in younger adult patients with non-acute promyelocytic AML

**DOI:** 10.3389/fimmu.2024.1321126

**Published:** 2024-04-22

**Authors:** Qi Hou, Penglin Wang, Xueting Kong, Junjie Chen, Chao Yao, Xiaodan Luo, Yangqiu Li, Zhenyi Jin, Xiuli Wu

**Affiliations:** ^1^ Institute of Hematology, Medical Laboratory Center, School of Medicine, Jinan University, Guangzhou, China; ^2^ Department of Pathophysiology, School of Medicine, Jinan University, Guangzhou, China; ^3^ Department of Hematology, Huazhong University of Science and Technology Union Shenzhen Hospital (Nanshan Hospital), Shenzhen, China; ^4^ Department of Hematology, The Fifth Affiliated Hospital of Guangzhou Medical University, Guangzhou, China; ^5^ Key Laboratory of Viral Pathogenesis and Infection Prevention and Control (Jinan University), Ministry of Education, Guangzhou, China; ^6^ Department of Pathology, School of Medicine, Jinan University, Guangzhou, China

**Keywords:** γδ T cells, younger AML, TIGIT, memory, prognosis

## Abstract

**Introduction:**

γδ T cells recognize and exert cytotoxicity against tumor cells. They are also considered potential immune cells for immunotherapy. Our previous study revealed that the altered expression of immune checkpoint T-cell immunoreceptor with immunoglobulin and ITIM domain (TIGIT) on γδ T cells may result in immunosuppression and is possibly associated with a poor overall survival in acute myeloid leukemia (AML). However, whether γδ T-cell memory subsets are predominantly involved and whether they have a relationship with clinical outcomes in patients with AML under the age of 65 remain unclear.

**Methods:**

In this study, we developed a multicolor flow cytometry-based assay to monitor the frequency and distribution of γδ T-cell subsets, including central memory γδ T cells (T_CM_ γδ), effector memory γδ T cells (T_EM_ γδ), and T_EM_ expressing CD45RA (T_EMRA_ γδ), in peripheral blood from 30 young (≤65 years old) patients with newly diagnosed non-acute promyelocytic leukemia (also known as M3) AML (AMLy-DN), 14 young patients with AML in complete remission (AMLy-CR), and 30 healthy individuals (HIs).

**Results:**

Compared with HIs, patients with AMLy-DN exhibited a significantly higher differentiation of γδ T cells, which was characterized by decreased T_CM_ γδ cells and increased T_EMRA_ γδ cells. A generally higher TIGIT expression was observed in γδ T cells and relative subsets in patients with AMLy-DN, which was partially recovered in patients with AMLy-CR. Furthermore, 17 paired bone marrow from patients with AMLy-DN contained higher percentages of γδ and TIGIT+ γδ T cells and a lower percentage of T_CM_ γδ T cells. Multivariate logistic regression analyses revealed the association of high percentage of TIGIT+ T_CM_ γδ T cells with an increased risk of poor induction chemotherapy response.

**Conclusions:**

In this study, we investigated the distribution of γδ T cells and their memory subsets in patients with non-M3 AML and suggested TIGIT+ T_CM_ γδ T cells as potential predictive markers of induction chemotherapy response.

## 1 Introduction

Acute myeloid leukemia (AML) is a prevalent form of leukemia in adults, which is characterized by the disruption of the normal hematopoiesis process. This results in the accumulation of immature myeloid cells in both the bone marrow (BM) and peripheral blood (PB) (1). Among them, the recurrence rate of acute myeloblastic leukemia with maturation (M2) and acute monocytic leukemia (M5) types is high (2). In general, acute promyelocytic leukemia (APL, also known as M3) is a unique subtype of AML, which is a highly curable cancer with long-term survival exceeding 90% (3). Excluding M3 AML, the complete remission (CR) of AML following chemotherapy induction is estimated to be approximately 70%–80%. However, long-term overall survival (OS) and disease-free survival rates can be discouragingly low (4). Furthermore, advanced age represents a crucial adverse prognostic factor of AML (5). Given their poor outcomes, considerable attention has been focused on elderly patients (over 65 years of age); however, research specifically targeting younger adults (less than 65 years) with non-M3 AML is relatively sparse (6).

Immune escape is also a crucial factor contributing to disease progression and poor clinical outcomes of AML (7). Immune escape is primarily attributed to the downregulation of immune cell function and exhaustion. It includes the upregulation of immune checkpoint (IC) receptors and a high expression of IC ligands on tumor cells (8). Our previous work demonstrated that T-cell immune inhibitory receptors, such as program death-1 (PD-1), T-cell immunoreceptor with immunoglobulin and ITIM domain (TIGIT), T-cell immunoglobulin mucin 3 (Tim-3), and T lymphocyte activation gene-3 (LAG-3), show increased expression in T cells from patients with newly diagnosed AML (AMLy-DN) and those with relapsed AML. In addition, increased IC expression has been associated with clinical outcomes, which suggests that the dysregulation of these immune inhibitory receptors may contribute to immune escape and poor prognosis of AML (9, 10). TIGIT is predominantly expressed on memory T cells, regulatory T cells (Tregs), and natural killer (NK) cells in humans. Studies have shown that the increased expression of TIGIT on immune cells is associated with functional exhaustion (11). The upregulation of TIGIT on immune cells, particularly T cells, has been implicated in immune escape mechanisms and can contribute to poor clinical outcomes of various diseases, including AML (10, 12). In addition, T cells within the BM immunosuppressive tumor microenvironment (TME) in AML often exhibit functional exhaustion and a deregulated innate and adaptive immune response (13).

As a minor subset of lymphocytes, human γδ T cells account for approximately 2%–10% of CD3+ T cells in PB and exhibit non-major histocompatibility complex (non-MHC)-restricted recognition of tumor antigens (14, 15). Upon activation in the periphery, γδ T cells exhibit remarkably diverse effector functions associated with immune response and a potent cytotoxic activity via elevated levels of CD107a expression and interferon (IFN)-γ cytokine production, granzyme B, and perforin secretion (16, 17). Previously, we highlighted γδ T-cell-based immunotherapy as a highly promising strategy in cancer immunotherapy (18). However, accumulating evidence indicates the diverse structural and functional heterogeneity among γδ T cells, which is associated with their distinct roles in cancer immunity (19). Importantly, our previous data have demonstrated a potential correlation between the expression profile of coinhibitory and costimulatory receptors on γδ T cells and distinct clinical outcomes in patients with AML (20, 21). Similar to αβ T cells, γδ T cells comprise various subtypes based on their diverse functions (22). Effector γδ T cells exert an antitumor effect through various pathways, and regulatory or inhibitory γδ T cells play a pivotal role in immune homeostasis and stable immune tolerance (14). In contrast, γδ T cells in adult PB exhibit various phenotypic markers commonly associated with memory cells; these cells display heterogeneity, which enables the identification of various distinct cell subsets based on their functional markers (23). Circulating γδ T cells are classified as naïve type (T_N_ γδ, antigen inexperienced) and memory γδ T cells (antigen experienced). Differential coexpressions of CD45RA and CD27 can be utilized to identify distinct subsets of memory γδ T cells, including central memory T cells (T_CM_ γδ), effector memory T cells (T_EM_ γδ), and T_EM_ expressing CD45RA (T_EMRA_ γδ), which represent various stages of differentiation. Following antigen stimulation, T_CM_ γδ gain the ability to maintain long-term immune memory and rapidly mediate immune response (24). The T_EM_ and T_EMRA_ γδ subsets predominantly exist at inflammatory sites and exert immediate effects via the secretion of cytokines and cytotoxicity (25). Our laboratory data indicate the dramatic effect of aging on T-cell subsets (26). Xu et al. in our laboratory observed a considerable decrease in the frequency of T_CM_ γδ in CD8+ T cells with an increase in differentiated T_EM_ γδ, particularly in younger patients with AML. This condition may be associated with suppressed T-cell immunity and diminished antileukemia capacity (27).

Several ongoing clinical trials are currently investigating the potential of γδ T cells in the adoptive therapy of AML and other hematologic malignancies (28, 29). However, γδ T-cell approaches exhibit limitations in terms of expansion and lifespan *in vivo*. Under unfavorable conditions, their relative plasticity can lead to phenotypes that are detrimental to the host. Our previous study demonstrated a correlation between the high frequencies of TIGIT+Foxp3+ and TIGIT+CD226− γδ T subsets and poor survival outcomes in patients with AML (30). Nevertheless, no discussion has been conducted on future perspectives regarding the phenotypic and functional characteristics of memory γδ T cells, particularly those observed in younger patients with AML. For the achievement of this goal, we aimed to define the distinct features of γδ T-cell memory phenotype in PB and BM from patients with non-M3 AML under 65 years old (referred to as AMLy-DN cells in this study) while exploring associations between the immunosuppression status and clinical outcomes among different patients. This comprehensive study will facilitate the cautious application of γδ T cells for patients with AML in the future.

## 2 Materials and methods

### 2.1 Samples

PB was collected from 37 patients with AMLy-DN, including 23 male and 14 female patients, with a median age of 50 years (range: 21–65 years). There were 30 patients with AMLy-DN for FACS analysis and 9 patients for cytokine secretion detection (only two of them were used for both FACS and cytokine secretion). In addition, PB samples were obtained from 14 patients with AML in CR (AMLy-CR), which consisted of 5 male and 9 female patients with a median age of 36 years (range: 24–62 years), 5 of whom are paired to the samples of AMLy-DN. Furthermore, BM samples were collected from 16 patients with AMLy-DN, namely, 12 male and 4 female patients with a median age 49 years (range: 23–65 years). We included 30 healthy individuals (HIs), which comprised 18 male and 12 female patients with a median age of 45 years (range: 19–65 years), as controls. All specimens were collected between October 2020 and March 2022. Three patients voluntarily withdrew from the hospital, two of whom refused therapies, and the other one was transferred to another hospital due to COVID-19. Consequently, prognosis analysis was performed on 27 patients with AMLy-DN. **Table 1** presents the corresponding clinical details. All participants provided informed consent. The experimental protocol for all studies was approved by the Ethics Committee of the First Affiliated Hospital of Jinan University. All procedures accorded with the guidelines set forth by the Medical Ethics Committees of the Health Bureau of Guangdong Province in China.

**Table 1:** Clinical characteristics of patients with AML.

Variables	Patients (total *n* = 37)
Age, mean ± SD, years	44 ± 15
Gender, *n* (%)	
Female	14 (37.8)
Male	23 (62.2)
WBC (×10^9^/L), (median; range)	10.1 (1–318.4)
BM blast cells (%), (median; range)	66 (20.5–97.5)
Risk stratification, *n* (%)	
Low	3 (8.1)
Intermediate	9 (24.3)
High	17 (45.9)
Unknown	8 (21.6)
Genotype abnormality, *n* (%)	
No	3 (8.1)
Yes	26 (70.3)
Unknown	8 (21.6)
*FLT3* mutation, *n* (%)	
No	20 (54.1)
Yes	9 (24.3)
Unknown	8 (21.6)
Subtype, *n* (%)	
M1	3 (8.1)
M2	12 (32.4)
M4	5 (13.5)
M5	10 (27.0)
Unclassified	7 (18.9)
Cytogenetic abnormality, *n* (%)	
Normal	8 (21.6)
Abnormal	11 (29.7)
Unknown	18 (48.6)
Allo-HSCT, *n* (%)	
Yes	8 (21.6)
No	22 (59.5)
Unknown	7 (18.9)
Follow-up, median (range), days	565 (83,952)
Status	
Alive	36 (97.3)
Dead	1 (2.7)

AML, acute myeloid leukemia patients under 65 years; SD, standard deviation; WBC, white blood cell; BM, blast cells, bone marrow blast cells; allo-HSCT, allogeneic hematopoietic stem cell transplantation.

### 2.2 Flow cytometry

The following monoclonal antibodies were used for cell-surface staining in accordance with the manufacturer’s instructions: CD3-APC/Cy7, TCR γδ-PE, CD27-PE/Cyanine 7, CD45RA-BV510, and TIGIT-BV421. In brief, 300 µL of PB from each patient was collected in a tube, and 5 µL per antibody was added to each tube for 20 min at room temperature in the dark. RBC Lysis Buffer was used to lyse erythrocytes for 10 min in the dark. The cells were completely washed afterward with phosphate buffer saline (GenXion, China).

For intracellular cytokine expression, cells were stimulated with phorbol myristate acetate (PMA, 0.05 μg/mL, Sigma-Aldrich, Germany) and brefeldin A (BFA, 10 μg/mL, BD Biosciences, USA) at 37°C for 5 h. Cells were stained with CD3 APC/Cy7 (clone SK7, BioLegend, USA), TCR γ/δ Percp-Cyanine 5.5 (clone B1, BioLegend, USA), and CD107a PE (clone SK7, BD Biosciences, USA), then fixed and permeabilized, followed by intracellular staining with IFN-γ-PE-Cy7 (clone 4S.B3, BioLegend, USA), perforin-Alexa-Fluor-647 (clone dG9, BioLegend, USA), and GZMB-Pacific blue (clone GB11, BioLegend, USA). Cells were acquired on a BD FACS VERSE flow cytometer (BD Biosciences, USA) and analyzed by FlowJo 10.5.3 software.

### 2.3 Statistical analysis

After the Shapiro–Wilk test revealed that the data were not normally distributed, the Mann–Whitney *U*-test was conducted to analyze statistical differences between the two groups. Paired samples were assessed via Wilcoxon matched-pair signed-rank test statistics. Pearson correlation analysis was performed to determine the correlation between the frequencies of TIGIT+ γδ T and γδ T memory cell subsets in each group. Binary logistic regression analysis was employed to investigate associations between the expression proportions of γδ T-cell subsets and clinical outcomes of patients with AML, and univariate analysis was used to select significant variables included in a multivariate analysis model. SPSS 25.0 and GraphPad Prism 8.4 were used in statistical analyses, with *p* ≤ 0.05 considered statistically significant.

### 2.4 Manuscript writing

The entire paper, including the Introduction and Materials and Methods sections, was polished and grammatically corrected by ShineWrite.com.

## 3 Results

### 3.1 γδ T cells shift toward effector memory and T_EMRA_ phenotype in PB from patients with AML

**Figure 1:**
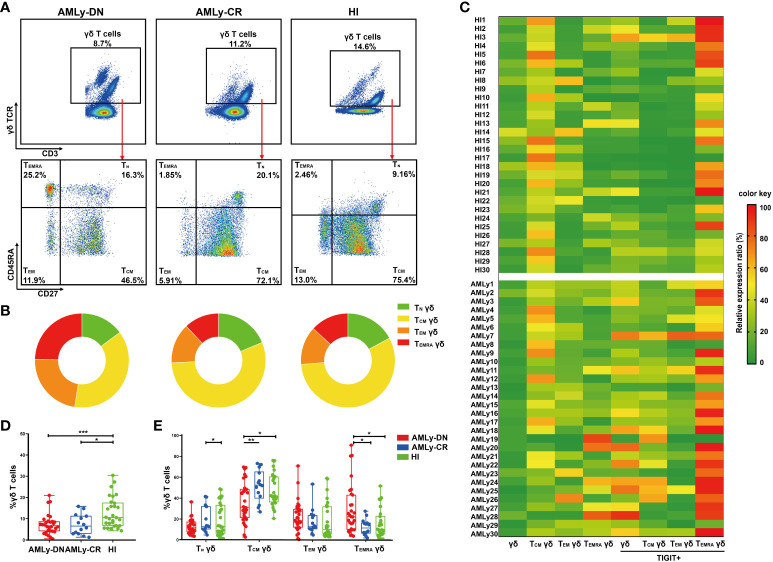
Significant reduction of γδ T_CM_ cells accompanied by an enrichment of γδ T_EMRA_ cells in patients with AMLy-DN. **(A)** Plots from AMLy-DN and AMLy-CR representative patients and a representative HI. γδ T cells were gated within the CD3 high population against the expression of γδ TCR. Then, within such γδ T+ population, CD45RA and CD27 T subsets were identified (red arrows). γδ T cells were differentiated into four memory subsets based on the expression of CD27 and CD45RA: T_N_ γδ T cells (CD27+CD45RA+), T_CM_ γδ T cells (CD27+CD45RA−), T_EM_ γδ T cells (CD27−CD45RA−), and T_EMRA_ γδ T cells (CD27−CD45RA+). **(B)** Summary of the distribution of four subpopulations in γδ T cells in patients with AMLy and HIs, as percentages from the averages of each group in pie charts. **(C)** Heatmap shows the frequency (low to high, respectively, from green to red shades) of TIGIT and memory cell subpopulations of γδ T cells in patients with AMLy-DN and HIs. **(D)** The distribution of γδ T cells in patients with AMLy and HIs (AMLy-DN: *n* = 30, AMLy-CR: *n* = 14, HI: *n* = 30). **(E)** Frequency of the T_N_, T_CM_, T_EM_, and T_EMRA_ γδ T-cell subsets in patients with AMLy and HIs (AMLy-DN: *n* = 30, AMLy-CR: *n* = 14, HI: *n* = 30). *p < 0.05, **p < 0.01, and ***p < 0.001.

In this study, we assessed the distribution of γδ T cells and their subsets in PB from patients with AMLy-DN (*n* = 30), those with AMLy-CR (*n* = 14), and HIs (*n* = 30) via multicolor flow cytometry. The γδ T cells were further categorized into four subgroups based on the expression patterns of CD27 and CD45RA: T_N_ γδ cells (CD27+CD45RA+), T_CM_ γδ cells (CD27+CD45RA−), T_EM_ γδ cells (CD27−CD45RA−), and T_EMRA_ γδ cells (CD27−CD45RA+) (24). **Figures 1A, B** illustrate the gating strategy for the identification of these subsets. Heatmap analysis revealed significant differences in the frequencies of TIGIT expression and memory subset distribution among different groups (**Figure 1C**). Our findings demonstrated the decreased proportion of total γδ T cells in PB from the AMLy-DN (*p* = 0.001) and AMLy-CR groups (*p* = 0.011) compared with that in HIs (**Figure 1D**; **Table 2**). Moreover, the percentage of T_CM_ γδ cells showed a significant reduction within PB samples from patients with AMLy-DN compared with HIs (*p* = 0.024). Conversely, the T_EMRA_ γδ subset proportions exhibited a marked increase (*p* = 0.010) (**Figure 1E**; **Table 2**). However, no significant difference was detected between HIs and patients with AMLy-DN regarding T_EM_ γδ (*p* = 0.077) and T_N_ γδ (*p* = 0.515) cells. In contrast to HIs, where the central memory subset predominated over other subsets (T_CM_ γδ > T_EM_ γδ > T_EMRA_ γδ; **Table 2**), our results indicate that patients with AMLy-DN exhibited an altered pattern characterized by increased proportions of the T_EMRA_ γδ subset relative to the T_EM_ γδ population (T_CM_ γδ > T_EMRA_ γδ > T_EM_ γδ; **Table 2**). Thus, changes in the γδ T-cell subsets primarily involved memory subsets. Following chemotherapy of patients with AMLy-CR, we observed a significant increase in the percentage of T_CM_ γδ cells (*p* = 0.008), accompanied with a corresponding decrease in T_EMRA_ γδ cells (*p* = 0.008), compared with the AMLy-DN group (**Figure 1E**). No significant difference was observed between HIs and AMLy-CR groups, and AMLy-CR showed the following pattern: T_CM_ γδ > T_EM_ γδ > T_EMRA_ γδ (**Table 2**). These findings suggest that the shift from T_CM_ γδ to an elevated proportion of differentiated T_EMRA_ γδ cells may be attributed to dysfunctional γδ T cells in patients with AMLy-DN.

**Table 2:** Comparison of percentages (with IQR) of γδ T cell and its memory subsets in patients with AMLy-DN and HIs.

	HIs	DN	CR	*p*-value		
				DN vs. HIs	DN vs. CR	CR vs. HIs
γδ T cells	10.60 (7.00, 17.68)	7.14 (4.22, 8.43)	6.51 (3.13, 10.95)	0.001	0.940	0.011
T_N_ γδ	12.75 (6.18, 32.08)	11.60 (7.65, 17.23)	13.00 (10.30, 29.43)	0.515	0.217	0.597
T_CM_ γδ	42.05 (36.80, 60.50)	31.25 (21.80, 48.53)	52.45 (42.68, 62.23)	0.024	0.008	0.496
T_EM_ γδ	9.81 (6.09, 30.68)	18.15 (12.65, 29.13)	13.00 (10.38, 23.00)	0.077	0.178	0.420
T_EMRA_ γδ	9.79 (5.38, 21.45)	22.05 (9.89, 40.65)	11.40 (6.55, 16.00)	0.010	0.008	0.830

DN, patients with newly diagnosed AML under 65 years; CR, patients with AML with complete remission; HIs, healthy individuals.

### 3.2 High TIGIT expression in γδ T-cell subsets in patients with AML

**Figure 2:**
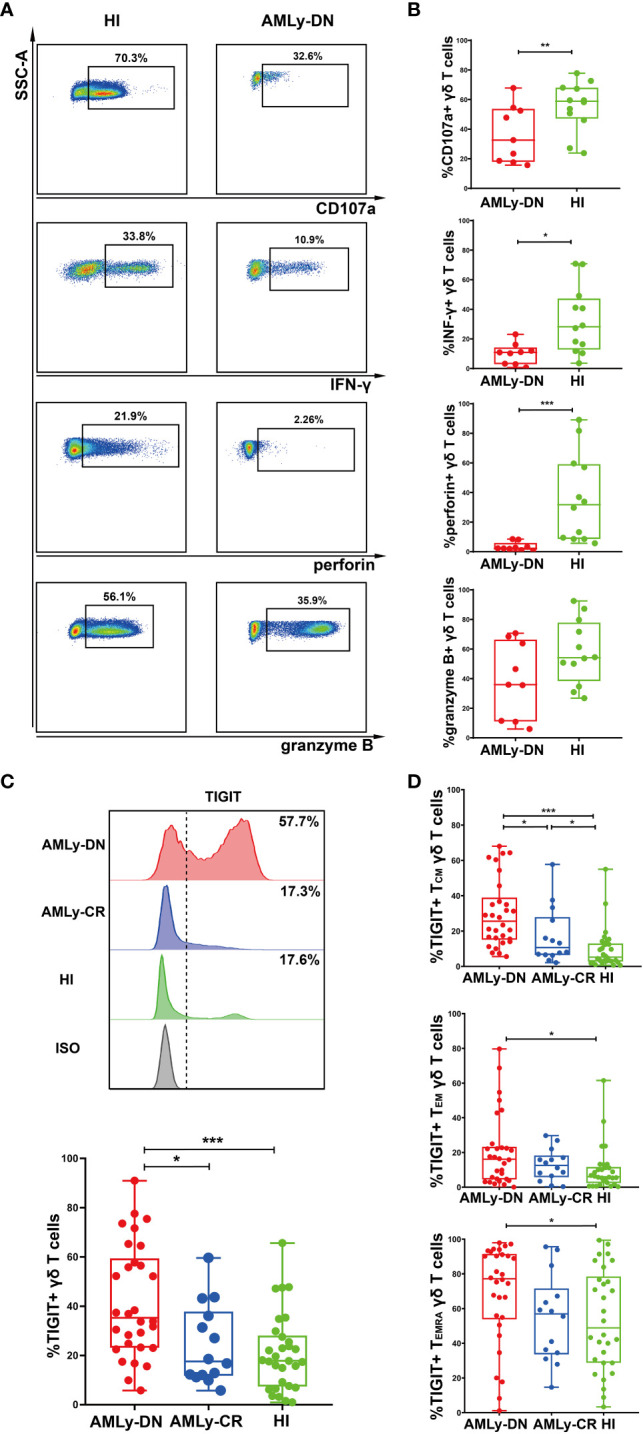
Decreased cytokine responses and high TIGIT expression in γδ T cells from patients with AMLy-DN. **(A)** Flow cytometric analysis shows the ability of γδ T cells to secrete cytokines. FACS plots display a representative AMLy-DN patient compared to an HI. **(B)** Statistical analysis of CD107a and cytokine responses of γδ T cells derived from multiple patients with AMLy-DN and HIs (AMLy-DN: *n* = 9, HI: *n* = 12). **(C)** The flow-cytometry analysis detected an increase in the frequency of TIGIT-expressing γδ T cells in the AMLy-DN compared with AMLy-CR and HIs. Data from representative AMLy-DN (red) and AMLy-CR (blue) patients, and HI (green), in comparison to the isotype control (HI was stained with isotype control antibody; gray). The distribution of TIGIT+ γδ T cells in patients with AMLy and HIs (AMLy-DN: *n* = 30, AMLy-CR: *n* = 14, HI: *n* = 30). **(D)** Frequency of TIGIT in the T_CM_, T_EM_, and T_EMRA_ γδ T-cell populations in patients with AMLy and HIs (AMLy-DN: *n* = 30, AMLy-CR: *n* = 14, HI: *n* = 30). *p < 0.05, **p < 0.01, and ***p < 0.001.

The intracellular cytokine secretion by γδ T cells from PB was also examined in patients with AMLy-DN (*n* = 9) and HIs (*n* = 15). Flow cytometry analysis revealed significant decreases in the proportions of CD107a, IFN-γ, and perforin on γδ T cells in patients with non-M3 AMLy-DN compared with HIs [CD107a: 59.50% (HIs) versus 32.60% (AMLy-DN), *p* = 0.008; IFN-γ: 27.30% (HIs) versus 10.90% (AMLy-DN), *p* = 0.025; perforin: 20.70% (HIs) versus 2.26% (AMLy-DN), *p* < 0.001]. However, the proportion of granzyme B on γδ T cells from patients with AMLy-DN was similar to that observed in HIs [54.50% (HIs) versus 35.90% (AMLy-DN), *p* = 0.108] (**Figures 2A, B**). Our findings reveal the impaired cytotoxic cytokine production and dysfunctional characteristics of γδ T cells derived from patients with AML.

We further aimed to investigate whether the increased proportion of TIGIT expression on γδ T-cell subsets contributed to the observed dysfunction in patients with AML. Our results demonstrate a significantly increased percentage of TIGIT+ γδ T cells in patients with AMLy-DN compared with those in HIs (*p* < 0.001) and AMLy-CR (*p* = 0.014) (**Figure 2C**; **Table 3**). Furthermore, TIGIT showed an elevated expression on all γδ T-cell memory subsets in patients with AMLy-DN compared with HIs (TIGIT+ T_CM_ γδ: *p* < 0.001; TIGIT+ T_EM_ γδ: *p* = 0.029; TIGIT+ T_EMRA_ γδ: *p* = 0.034) (**Figure 2D**; **Table 3**). In patients with AMLy-CR, the expression of TIGIT within T_CM_ γδ cell was significantly lower than that in patients with AMLy-DN (*p* = 0.013). However, no statistically significant difference was detected in the percentages of T_EM_ γδ (*p* = 0.420) and T_EMRA_ γδ cell subsets (*p* = 0.078) between patients with AMLy-DN and those with AMLy-CR (**Figure 2D** and **Table 3**). Notably, only the expression of TIGIT+ T_CM_ was higher in patients with AMLy-CR compared with the HI group (*p* = 0.045), whereas other memory subsets from CR patients showed similar percentages (TIGIT+ T_EM_ γδ: *p* = 0.115; TIGIT+ T_EMRA_ γδ: *p* = 0.930).

**Table 3:** Comparison of percentages (with IQR) of positivity of TIGIT on γδ T cells in patients with AMLy-DN and HIs.

	HIs	DN	CR	*p*-value		
				DN vs. HIs	DN vs. CR	CR vs. HIs
TIGIT+ γδ	17.85 (7.92, 26.98)	35.30 (23.58, 57.38)	17.60 (12.05, 34.83)	<0.001	0.014	0.614
TIGIT+ T_CM_ γδ	5.26 (2.94, 12.65)	25.55 (15.65, 36.28)	10.65 (6.64, 23.58)	<0.001	0.013	0.045
TIGIT+ T_EM_ γδ	6.04 (2.82, 11.18)	16.10 (4.67, 22.63)	12.55 (6.91, 16.70)	0.029	0.420	0.115
TIGIT+ T_EMRA_ γδ	48.85 (29.18, 76.40)	77.20 (55.15, 90.90)	57.00 (34.95, 66.28)	0.034	0.078	0.930

DN, patients with newly diagnosed AML under 65 years; CR, patients with AML with complete remission; HIs, healthy individuals.

AML is a heterogeneous disease, and its prognosis varies significantly among different subtypes and genetic alterations (31). Therefore, we assessed and compared the distribution of γδ T cells and their memory subsets between the AML-M2 (*n* = 9) and AML-M5 (*n* = 10) subtypes. The frequency of γδ T cells tended to be higher in AML-M2 more than AML-M5 (*p* = 0.666) (**Supplementary Figure 1A**). Moreover, the AML-M2 group showed a lower frequency of TIGIT+ γδ subsets compared with the M5 group (*p* = 0.031) (**Supplementary Figure 1B** and **Supplementary Table 1**). Most notably, the response rate to induction therapy and CR rate reached 77.78% (7/9) in the M2 group and 40% (4/10) in the M5 group (**Supplementary Figure 1C**). Thus, our data reveal distinct distribution patterns of TIGIT on γδ T cells, correlating with favorable prognosis subtypes AML-M2 and AML-M5.

### 3.3 High TIGIT+ γδ and TIGIT+ γδ T_CM_ γδ cells in the BM of patients with AMLy-DN

**Figure 3:**
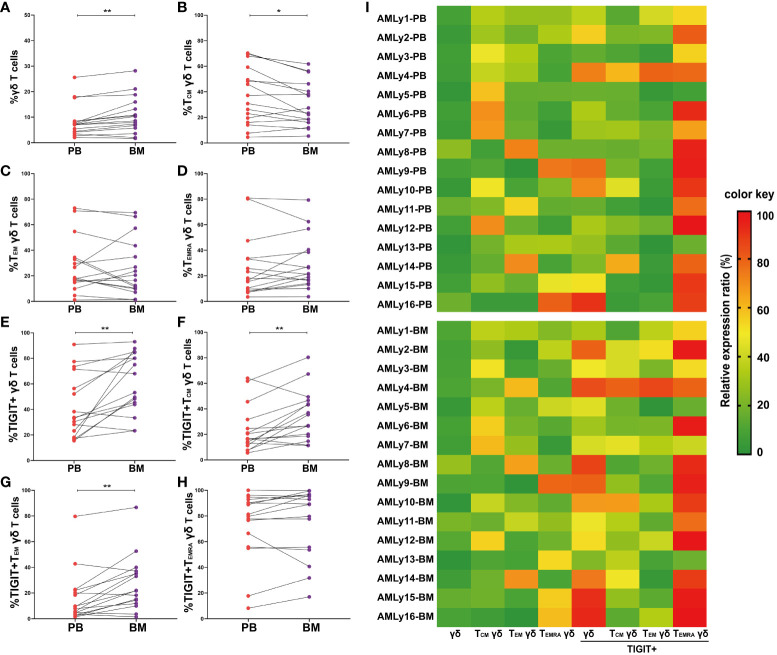
Significantly higher TIGIT expression in γδ T cells from the bone marrow of patients with AMLy-DN, relative to matched peripheral blood samples. **(A–D)** Pairwise comparisons of memory γδ cell subsets between PB and BM in AMLy-DN. (PB: *n* = 16, BM: *n* = 16). **(E–H)** Paired statistical analysis of TIGIT expression in γδ T cells and relative memory subsets between PB (red) and BM (violet) derived from AMLy-DN. **(I)** Heatmap shows the frequency of TIGIT and memory cell subpopulations of γδ T cells in the PB and BM of patients with AMLy-DN. *p < 0.05, **p < 0.01.

The immunosuppressive TME shields malignant hematopoietic stem cells from immune surveillance and potentially contributes to leukemia relapse. To investigate the effect of TME on γδ T-cell subset distribution in patients with AMLy-DN, we collected 16 pairs of PB and BM samples at diagnosis and compared the distributions of memory γδ T-cell subsets (**Figures 3A–H** and **Supplementary Table 2**). We observed higher proportions of total γδ T cells (*p* = 0.002; **Figure 3A**) and TIGIT+ γδ T cells (*p* = 0.004; **Figure 3E**) in BM than in PB, which suggests that inhibitory receptor expression may affect the function of these cells in various compartments. In terms of memory subsets, a significantly low percentage of T_CM_ γδ cells was discovered in BM (*p* = 0.023; **Figure 3B**). However, other subsets showed no significant changes compared with the corresponding PB samples (T_EM_ γδ: *p* = 0.438; T_EMRA_ γδ: *p* = 0.255; respectively, **Figures 3C, D**). Interestingly, wide variations in the expression levels of inhibitory receptors were observed among different subsets. The results show significantly increased TIGIT+ T_CM_ γδ (*p* = 0.007; **Figure 3F**) and TIGIT+ T_EM_ γδ T cells (*p* = 0.003; **Figure 3G**) in the BM of patients with AMLy-DN compared with PB. However, no significant difference was observed for TIGIT+ T_EMRA_ γδ (*p* = 0.140; **Figure 3H**). Heatmap analysis suggests that the influence of TME on memory T-cell populations may be more pronounced for the T_CM_ subsets (**Figure 3I**; note the transition from mainly green to yellow and darker orange/red shadows when comparing PB to BM for TIGIT+ γδ total T cells and TIGIT+ γδ T_CM_, as examples).

### 3.4 Increased frequencies of TIGIT+ T_CM_ γδ T cells are associated with poor response to chemotherapy

We collected clinical data from 30 patients with AMLy-DN to investigate the potential correlation between the phenotypes of γδ T-cell subsets and clinical response in patients with AML. After the exclusion of 3 patients who declined therapy and left the hospital voluntarily, we analyzed data from 27 patients who underwent chemotherapy following diagnosis. Based on follow-up after induction chemotherapy, patients with AMLy-DN were categorized into two groups: those who achieved CR (17 cases) and those who were non-CR (NCR) (10 cases). Univariate and multivariate logistic regression analyses were conducted to assess the proportions of γδ T and its subsets and other factors, such as gender, age, white blood cells, and BM blast cells in patients with AMLy-DN. The results of univariate logistic regression analysis reveal that those high frequencies of TIGIT+ γδ cells [hazard ratio (HR) = 1.044, 95% confidence interval (CI): 1.001,1.089, *p* = 0.043], TIGIT+ T_CM_ γδ cells (HR = 1.081, 95% CI: 1.016,1.150, *p* = 0.014), and TIGIT+ T_EMRA_ γδ cells (HR = 1.079, 95% CI: 1.006,1.159, *p* = 0.033) were independent risk factors against the attainment of CR in patients with AMLy-DN with non-M3 subtype disease. Notably, multivariate logistic regression analysis demonstrated TIGIT+ T_CM_ γδ cells (HR = 1.081, 95% CI: 1.016,1.151, *p* = 0.014) as an independent risk factor for worse prognosis in patients with non-M3 subtype AMLy-DN, and it can potentially serve as a biomarker for risk stratification (**Table 4**). We further sought to validate the influence of TIGIT expression on γδ T-cell subsets by comparing pre- and postinduction chemotherapy percentages among five selected participants in our study. The findings indicate decreases in TIGIT+ T_CM_ γδ and TIGIT+ T_EM_ in patients with AMLy who achieved CR status after the first cycle of chemotherapy (TIGIT+ T_CM_ γδ: *p* = 0.043; TIGIT+ T_EM_: *p* = 0.043, respectively) (**Supplementary Figure 1D**), suggesting a potential correlation with blast elimination. Whether TIGIT expression on memory subsets can be used to predict the survival of patients with AMLy needs to be confirmed in future studies.

**Table 4:** Univariate and multivariate regression analysis of γδ T-cell subsets in AMLy-DN.

Variables	Univariate regression		Multivariate regression	
	HR (95% CI)	*p*-value	HR (95% CI)	*p*-value
Gender				
Female	Reference			
Male	0.381 (0.073,1.992)	0.253		
Age, years	1.025 (0.969,1.084)	0.387		
Subtype				
M1	Reference	0.563		
M2	0.400 (0.026,6.176)	0.512		
M4	0.229 (0.029,1.774)	0.158		
M5	–	0.999		
WBC,10^9^/L	1.005 (0.995,1.014)	0.354		
BM blast cell, %	1.012 (0.977,1.049)	0.504		
FLT3				
Normal	Reference	0.596		
FLT3_Mut	–	0.999		
Others	0.286 (0.026,3.196)	0.309		
CD3%	0.986 (0.951,1.021)	0.422		
γδ%	0.896 (0.715,1.123)	0.343		
T_N_ γδ%	1.018 (0.916,1.132)	0.738		
T_CM_ γδ%	0.960 (0.915,1.008)	0.099		
T_EM_ γδ%	1.002 (0.951,1.056)	0.933		
T_EMRA_ γδ%	1.025 (0.987,1.065)	0.199		
TIGIT+ γδ%	1.044 (1.001,1.089)	0.043		
TIGIT+ T_CM_ γδ%	1.081 (1.016,1.150)	0.014	1.081 (1.016,1.150)	0.014
TIGIT+ T_EM_ γδ%	1.028 (0.988,1.069)	0.173		
TIGIT+ T_EMRA_ γδ%	1.080 (1.006,1.159)	0.033		

HR, hazard ratio; CI, confidence interval; WBC, white blood cell; BM, bone marrow.

## 4 Discussion

The in-depth understanding of pathogenesis of M3 heralded the introduction of highly effective therapies targeting the mutant protein *PML-RARα* that drives the disease, which led to the chemotherapy-free approach in the treatment of almost all patients (32). Despite the improved genetic understanding of AML, excluding the M3 subtype, some of these studies have overlooked the effect of patient age. Younger patients with non-M3 AML represent a distinct group with specific needs and minimal survival improvement (33). Therefore, our present research primarily focused on discussing non-M3 AML in younger patients.

T-cell immunotherapy has been increasingly investigated in AML (16). Nevertheless, the redirection of pan CD3+ T cells to target leukemia blasts has demonstrated limited efficacy in clinical trials and is often accompanied with severe toxicity in patients with AML due to T-cell immune dysfunction (17, 34). *In vitro* and mouse model studies have shown the cytotoxic effects of γδ T cells on AML cells (35). Our previous study demonstrated a correlation between reduced levels of circulating γδ T cells and poor survival outcomes (36). In this study, the proportions of total γδ T cells from PB decreased in patients with AMLy-DN and AMLy-CR relative to HIs. Apart from the effect on proportions, some γδ T cells that are cytotoxic against tumor cells may be susceptible to immunosuppressive mechanisms. Multiple ICs are commonly expressed on T cells in hematologic malignancies. Consequently, researchers studying the applications of γδ T cells have shown interest in improving their antitumor potential while addressing the challenges posed by IC expression and its contribution to leukemia development and progression (37, 38). Our previous study revealed a correlation between the decreased frequency of γδ T cells and increased expression of PD-1+Foxp3+ γδ T-cell subsets, and this finding was associated with a poor OS (20). Consistent with these findings, our current study demonstrated the significantly high expression of TIGIT on γδ T cells and low production of CD107a, IFN-γ, and perforin by γδ T cells in patients with AMLy-DN, which suggests a diminished antileukemia effect. This result is in line with the observations of Song et al., who reported the upregulated expression of TIGIT on blood T cells from elderly HIs (39). Furthermore, the downregulation of TIGIT can potentially restore T-cell function and improve IFN-γ production in hematological malignancies (40). Gournay et al. reported the association of the elevated expression of TIGIT on various subsets of T cells with subsequent AML relapse (41). As a follow-up to this finding, we assessed the significantly increased expressions of TIGIT+CD226− and TIGIT+Foxp3+ γδ T-cell subsets in *de novo* patients with AML, which indicated the exhaustion and heterogeneity of T cells (30). Despite the considerable efficacy of blocking TIGIT in preclinical research and clinical trials for hematological malignancies, the distribution characteristics of TIGIT in γδ T cells from AML have not been comprehensively investigated (42, 43). Importantly, our results further suggest the need to explore the characterization of TIGIT+ γδ T cells, which may respond to immune checkpoint inhibitor (ICI) treatment, and identify subpopulations with a stronger response. Therefore, studies should consider checkpoint inhibitor-based immunotherapy aimed at reinvigorating the antitumor activity of T cells and successful treatment strategies involving γδ T cells to mitigate the aberrant activation of specific γδ T-cell subsets.

In addition, T-cell immune surveillance is a crucial host defense mechanism in the suppression of carcinogenesis (44). Notably, the establishment of T-cell memory serves as a pivotal process for the long-term protection against tumor elimination by the host’s immune system (45). Less-differentiated subsets of T_CM_ cells exhibit enhanced antitumor efficacy compared with the more-differentiated T_EM_ and T_EMRA_ cells (46, 47). Our previous study proposed several models for the phenotypic classification of human CD8+ T cells; hence, the classification of human γδ T cells in terms of CD27 and CD45RA markers is valuable for the identification of naïve and memory cells (26). Subsets of memory γδ T cells possess similar developmental potential and can be categorized into T_CM_, T_EM_, and T_EMRA_ γδ cells based on phenotypic markers and functional attributes (24). In this study, changes in the subsets of memory γδ T cells primarily involved a decrease in T_CM_ γδ cells and an increase in T_EMRA_ γδ cells from patients with AMLy-DN compared with those from HIs. Upon CR after induction chemotherapy, restored T_CM_ γδ cell frequency and decreased T_EMRA_ γδ cell frequency were observed in PB. This finding suggests that the capacity for T-cell immune surveillance may depend on the response to chemotherapy (48). We previously reported that the frequencies of stem cell memory T (T_SCM_) and T_CM_ on CD8+ T cells dramatically decreased together with increases in T_EM_ and terminal effector T cells in the AMLy-DN; however, these alterations persisted in patients who achieved CR after chemotherapy (27). A previous study has positioned CD8+ memory T cells and the T_CM_ cell subset in an intermediate position between naïve and effector cells (24). T_SCM_ and T_CM_ subsets demonstrate superior performance in adoptive cell immunotherapy despite the improved cytotoxic and cytokine-releasing potential of effector T-cell subsets (49). A research revealed the positive correlation between the infiltration of T_CM_ and T_EM_ with a favorable prognosis in Ewing sarcoma (50). Our results indicate T_CM_ cells as a representative of a predominant population that exhibits superior capabilities as mediators of recall responses to antigen challenges given their prolonged persistence, rapid activation, and migration. Importantly, the classical T_CM_ cell compartment is an ideal source of T cells for immunotherapy against malignancy, which occurs in the form of long-term control effectors and central memory pools.

TIGIT, an inhibitory receptor expressed on lymphocytes, is a crucial IC that can impede every step of cancer immunity (51). This study revealed the significantly higher expression of TIGIT on memory γδ T-cell subset distributed in the AMLy-DN cohort than in younger HIs, which indicates the preferential expression of TIGIT on memory γδ T cells and its similar distribution pattern with that detected in older AML groups. Although TIGIT+ γδ T cells did not differ in frequency between AMLy-CR and HIs, the percentage of TIGIT+ T_CM_ γδ were intermediate in between the levels found in healthy donors and patients with AMLy-DN. Patients with AMLy-CR and HIs exhibited significant differences in terms of the frequency of γδ T_CM_ cells and TIGIT expression on the γδ T_CM_, which suggests that etiology may affect the generation or treatment response of memory γδ T cells and thereby influence their immunity and clinical outcome. These findings suggest that TIGIT+ T_CM_ γδ cells may play a role in AMLy surveillance against tumor cells and can be targeted for ICI treatment. Given the unresolved question regarding the mechanisms involved in γδ T-cell differentiation, we aimed to gain insights into the processes underlying the complexity of differentiated T-cell populations. Our results show that the younger patients with AML with a high frequency of TIGIT+ T_CM_ γδ T cells had a low likelihood of remaining in remission. The frequency of TIGIT+ T_CM_ γδ and TIGIT+ T_EM_ γδ predominantly decreased after CR, and changes in other memory γδ T-cell subsets varied relatively. Therefore, evaluation of the frequency of TIGIT+ T_CM_ γδ T cells before and after treatment can provide important information on their efficacy. The capability of anti-TIGIT reagents to reverse the suppression of TIGIT+ T_CM_ γδ T cells can be used as potential control measures that can enhance the activity of transferred γδ T cells. Importantly, T_CM_ γδ cells in PB exhibit a long-term maintenance property, which results in their sustained and durable responses to ICI (52) treatment, and the enhanced response by T_CM_ is associated with the complete eradication of leukemia cells (53). The efficient physiological responses from T_CM_ γδ cells may confer advantages to their adoptively transferred effector cell progeny.

Our previous studies primarily focused on γδ T cells in the PB. However, increasing evidence suggests that an immunosuppressive microenvironment promotes the emergence of phenotypically and functionally impaired γδ T cells, which leads to immune evasion by leukemia cells. Recognizing the importance of BM as another immunological environment, we compared the distributions of γδ T cells and their subsets in PB and BM samples from patients with AMLy-DN to investigate the influence of TME. Significant variations were observed in each subset between PB and BM, with a notable abundance of γδ T cells and TIGIT+ γδ T-cell subsets in the BM of most patients with AMLy-DN. In addition, the BM contained a lower percentage of T_CM_ γδ T cells along with a higher percentage of TIGIT+ T_CM_ and TIGIT+ T_EM_ compared with PB. Our results demonstrate the possibly different effects of the AML BM microenvironment on T_CM_ γδ T-cell homing and their contribution to global γδ T-cell dysfunction. Our previous study also revealed the enrichment of CD8+ T cells expressing PD-1 and TIM-3 receptors in the BM of patients with AML compared with PB (9). The high expression of TIGIT is an essential indicator of the effectiveness of ICI treatment, with an adequate presence of tumor-infiltrating T cells in the TME serving as a prerequisite (54). T cells in normal BM predominantly exhibit a memory phenotype, particularly CD8+ T_CM_ cells, which suggests the varying effects of alterations in the leukemic BM niche on T_CM_ homing among different individuals with AML (55). These dysregulated features of γδ T cells resemble those observed in elderly individuals with human immunodeficiency, and thus, they potentially represent premature immunologic aging. Therefore, further gaining insights into the phenotype and function of disrupted γδ T cells in patients with AML can facilitate the development of novel immunotherapeutic strategies. However, our analysis was only based on limited clinical samples, and further investigation is needed to collect and track more samples to confirm the findings.

Recent publications of our team and others have reported the association of a high proportion of γδ T cells with improved OS (36). However, further investigation is needed to evaluate the predictive value of OS in the follow-up period. Moreover, cytogenetic abnormalities are a critical prognostic factor for AML. Nevertheless, given the presence of FMS-like tyrosine kinase 3 mutation in nine cases, statistical analysis becomes challenging. Our findings support the notion that γδ T cells may serve as suitable targets for therapeutic intervention or cell therapy. Given these issues, further research on the expansion of long-lived memory γδ T cells must be conducted.

## 5 Conclusions

**Figure 4:**
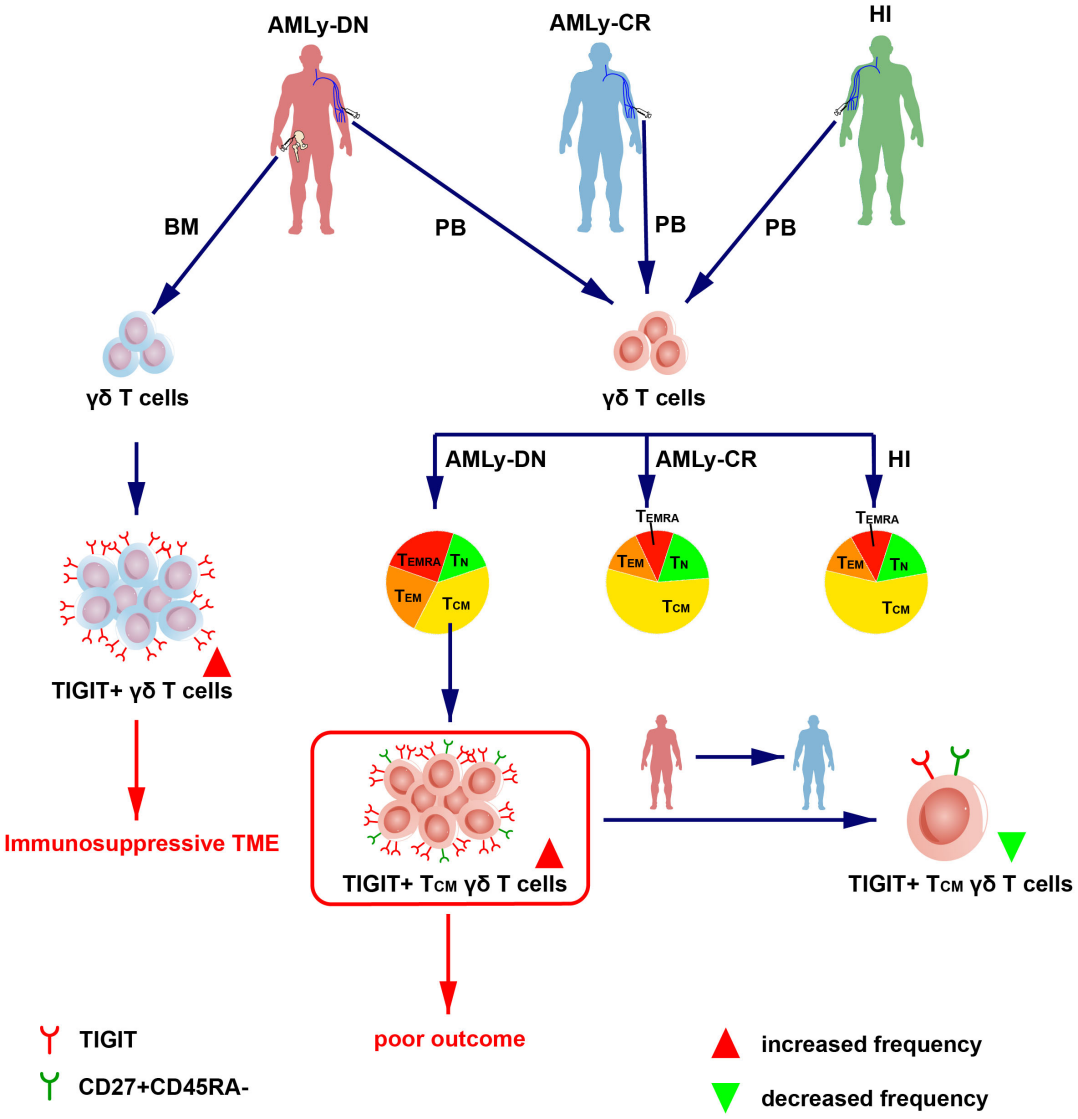
Overview of TIGIT changes in the γδ T-cell memory subsets in patients with AML. (Left) The high expression of TIGIT in γδ T cell in BM from patients with AMLy-DN may lead to immunosuppression of TME. (Right) The dendrogram shows the different distribution of γδ T-cell subsets from AMLy-DN, AMLy-CR, and HIs, and the high expression of TIGIT+ T_CM_ γδ T cell in patients with AMLy-DN may be related to poor outcome.

We initially characterized the distribution of γδ memory T-cell subsets in patients with non-M3 AML and used it to validate the relationship between AML prognosis and subsequent identification of a robust TIGIT+ T_CM_ γδ cell signature, which is significantly associated with AML prognosis (**Figure 4**). The findings have potential implications for guiding the selection and generation of optimal antileukemic T cells for adoptive immunotherapy, wherein the generated cells should acquire a T_CM_ phenotype with a low expression of TIGIT prior to transfer.

## Data availability statement

The original contributions presented in the study are included in the article/supplementary material, further inquiries can be directed to the corresponding authors.

## Ethics statement

The studies involving humans were approved by the Ethics Committee of the First Affiliated Hospital of Jinan University. The studies were conducted in accordance with the local legislation and institutional requirements. The participants provided their written informed consent to participate in this study.

## Author contributions

QH: Writing – original draft. PW: Writing – original draft. XK: Writing – original draft. JC: Writing – original draft. CY: Writing – original draft. XL: Writing – original draft. YL: Writing – review & editing. ZJ: Writing – review & editing. XW: Writing – review & editing.

## Supplementary material

Supplementary Figure 1 Different distribution pattern of TIGIT on γδ T cells between the AML-M2 and AML-M5. **(A)** The distribution of γδ T cells and their memory subsets in M2 and M5 subtypes. (M2: *n* = 9, M5: *n* = 10). **(B)** Frequency of TIGIT in the T_CM_, T_EM_, and T_EMRA_ γδ T-cell populations in M2 and M5 subtypes (M2: *n* = 9, M5: *n* = 10). **(C)** Distribution of CR or NR in M2 and M5 subtypes (M2: *n* = 9, M5: *n* = 10). **(D)** Pairwise comparisons of TIGIT in the γδ T-cell subsets by pre- and postinduction chemotherapy (pre-chemotherapy: *n* = 5, post-chemotherapy: *n* = 5). **p* < 0.05, ***p* < 0.01, and ****p* < 0.001.

## References

[1] DiNardo CD Erba HP Freeman SD Wei AH. Acute myeloid leukaemia. Lancet. (2023) 401:2073–86. doi: 10.1016/S0140-6736(23)00108-3 37068505

[2] He H Wang Z Yu H Zhang G Wen Y Cai Z. Prioritizing risk genes as novel stratification biomarkers for acute monocytic leukemia by integrative analysis. Discovery Oncol. (2022) 13:55. doi: 10.1007/s12672-022-00516-y PMC9247126 35771283

[3] Iyer SG Elias L StanChina M Watts J. The treatment of acute promyelocytic leukemia in 2023: Paradigm, advances, and future directions. Front Oncol. (2023) 12:1062524. doi: 10.3389/fonc.2022.1062524 36741714 PMC9889825

[4] Salhotra A Mei M. Acute promyelocytic leukemia: Update on risk stratification and treatment practices. Cancer Treat Res. (2021) 181:45–55. doi: 10.1007/978-3-030-78311-2_3 34626354

[5] Döhner H Estey E Grimwade D Amadori S Appelbaum FR Büchner T. Diagnosis and management of AML in adults: 2017 ELN recommendations from an international expert panel. Blood. (2017) 129:424–47. doi: 10.1182/blood-2016-08-733196 PMC5291965 27895058

[6] O'DwyerKFreyerDRHoranJT. Treatment strategies for adolescent and young adult patients with acute myeloid leukemia. Blood. (2018) 132:362–8. doi: 10.1182/blood-2017-12-778472 29895667

[7] Vago L Gojo I. Immune escape and immunotherapy of acute myeloid leukemia. J Clin Invest. (2020) 130:1552–64. doi: 10.1172/JCI129204 PMC7108895 32235097

[8] Abaza Y Zeidan AM. Immune checkpoint inhibition in acute myeloid leukemia and myelodysplastic syndromes. Cells. (2022) 11:2249. doi: 10.3390/cells11142249 35883692 PMC9318025

[9] Xu L Liu L Yao D Zeng X Zhang Y Lai J. PD-1 and TIGIT are highly co-expressed on CD8+ T cells in AML patient bone marrow. Front Oncol. (2021) 11:686156. doi: 10.3389/fonc.2021.686156 34490086 PMC8416522

[10] Tan J Tan H Li Y. Targeting TIM-3 for hematological Malignancy: latest updates from the 2022 ASH annual meeting. Exp Hematol Oncol. (2023) 12:62. doi: 10.1186/s40164-023-00421-2 37468979 PMC10357734

[11] Wang Y Zhang H Liu C Wang Z Wu W Zhang N. Immune checkpoint modulators in cancer immunotherapy: Recent advances and emerging concepts. J Hematol Oncol. (2022) 15:111. doi: 10.1186/s13045-022-01325-0 35978433 PMC9386972

[12] Chauvin JM Zarour HM. TIGIT in cancer immunotherapy. J Immunother Cancer. (2020) 8:e000957. doi: 10.1136/jitc-2020-000957 32900861 PMC7477968

[13] Yang L Li A Lei Q Zhang Y. Tumor-intrinsic signaling pathways: Key roles in the regulation of the immunosuppressive tumor microenvironment. J Hematol Oncol. (2019) 12:125. doi: 10.1186/s13045-019-0804-8 31775797 PMC6880373

[14] Sarkar I Pati S Dutta A Basak U Sa G. T-memory cells against cancer: Remembering the enemy. Cell Immunol. (2019) 338:27–31. doi: 10.1016/j.cellimm.2019.03.002 30928016

[15] de Vries NL van de Haar J Veninga V Chalabi M Ijsselsteijn ME van der Ploeg M. γδ T cells are effectors of immunotherapy in cancers with HLA class I defects. Nature. (2023) 613:743–50. doi: 10.1038/s41586-022-05593-1 PMC9876799 36631610

[16] Hao F Sholy C Wang C Cao M Kang X. The role of T cell immunotherapy in acute myeloid leukemia. Cells. (2021) 10:3376. doi: 10.3390/cells10123376 34943884 PMC8699747

[17] Zhang P Zhang G Wan X. Challenges and new technologies in adoptive cell therapy. J Hematol Oncol. (2023) 16:97. doi: 10.1186/s13045-023-01492-8 37596653 PMC10439661

[18] Zheng J Jiang X Zhao H Wang W Wu X Jin Z. Γδ T cells: A sparkling star for clinical immunotherapy. Explor Immunol. (2022) 2:540–57. doi: 10.37349/ei.2022.00066

[19] Li Y Li G Zhang J Wu X Chen X. The dual roles of human γδ T cells: Anti-tumor or tumor-promoting. Front Immunol. (2021) 11:619954. doi: 10.3389/fimmu.2020.619954 33664732 PMC7921733

[20] Zheng J Qiu D Jiang X Zhao Y Zhao H Wu X. Increased PD-1+Foxp3+ γδ T cells associate with poor overall survival for patients with acute myeloid leukemia. Front Oncol. (2022) 12:1007565. doi: 10.3389/fonc.2022.1007565 36591503 PMC9799959

[21] Jin Z Ye W Lan T Zhao Y Liu X Chen J. Characteristic of TIGIT and DNAM-1 expression on foxp3+ γδ T cells in AML patients. BioMed Res Int. (2020) 2020:4612952. doi: 10.1155/2020/4612952 32802845 PMC7403925

[22] Sallusto F Geginat J Lanzavecchia A. Central memory and effector memory T cell subsets: Function, generation, and maintenance. Annu Rev Immunol. (2004) 22:745–63. doi: 10.1146/annurev.immunol.22.012703.104702 15032595

[23] Mahnke YD Brodie TM Sallusto F Roederer M Lugli E. The who's who of T-cell differentiation: Human memory T-cell subsets. Eur J Immunol. (2013) 43:2797–809. doi: 10.1002/eji.201343751 24258910

[24] Dieli F Poccia F Lipp M Sireci G Caccamo N Di Sano C. Differentiation of effector/memory vdelta2 T cells and migratory routes in lymph nodes or inflammatory sites. J Exp Med. (2003) 198:391–7. doi: 10.1084/jem.20030235 PMC2194087 12900516

[25] Sallusto F Lenig D Förster R Lipp M Lanzavecchia A. Two subsets of memory T lymphocytes with distinct homing potentials and effector functions. Nature. (1999) 401:708–12. doi: 10.1038/44385 10537110

[26] Li M Yao D Zeng X Kasakovski D Zhang Y Chen S. Age related human T cell subset evolution and senescence. Immun Ageing. (2019) 16:24. doi: 10.1186/s12979-019-0165-8 31528179 PMC6739976

[27] Xu L Yao D Tan J He Z Yu Z Chen J. Memory T cells skew toward terminal differentiation in the CD8+ T cell population in patients with acute myeloid leukemia. J Hematol Oncol. (2018) 11:93. doi: 10.1186/s13045-018-0636-y 29986734 PMC6038290

[28] Vydra J Cosimo E Lesný P Wanless RS Anderson J Clark AG. A phase I trial of allogeneic γδ T lymphocytes from haploidentical donors in patients with refractory or relapsed acute myeloid leukemia. Clin Lymphoma Myeloma Leuk. (2023) 23:e232–9. doi: 10.1016/j.clml.2023.02.003 PMC10139146 36863897

[29] Nishimoto KP Barca T Azameera A Makkouk A Romero JM Bai L. Allogeneic CD20-targeted γδ T cells exhibit innate and adaptive antitumor activities in preclinical B-cell lymphoma models. Clin Transl Immunol. (2022) 11:e1373. doi: 10.1002/cti2.1373 PMC8809437 35136603

[30] Jin Z Lan T Zhao Y Du J Chen J Lai J. Higher TIGIT+CD226- γδ T cells in patients with acute myeloid leukemia. Immunol Invest. (2022) 51:40–50. doi: 10.1080/08820139.2020.1806868 32819181

[31] Catovsky D Tavares de Castro J. The classification of acute leukaemia (AL) and its clinical significance. Schweiz Med Wochenschr. (1983) 113:1434–7. 6359396

[32] Liquori A Ibañez M Sargas C Sanz MÁ Barragán E Cervera J. Acute promyelocytic leukemia: A constellation of molecular events around a single PML-RARA fusion gene. Cancers (Basel). (2020) 12:624. doi: 10.3390/cancers12030624 32182684 PMC7139833

[33] Visani G Chiarucci M Paolasini S Loscocco F Isidori A. Treatment options for acute myeloid leukemia patients aged <60 years. Front Oncol. (2022) 12:897220. doi: 10.3389/fonc.2022.897220 36276074 PMC9581198

[34] Tang L Wu J Li CG Jiang HW Xu M Du M. Characterization of immune dysfunction and identification of prognostic immune-related risk factors in acute myeloid leukemia. Clin Cancer Res. (2020) 26:1763–72. doi: 10.1158/1078-0432.CCR-19-3003 31911547

[35] Choi H Lee Y Hur G Lee SE Cho HI Sohn HJ. γδ T cells cultured with artificial antigen-presenting cells and IL-2 show long-term proliferation and enhanced effector functions compared with γδ T cells cultured with only IL-2 after stimulation with zoledronic acid. Cytotherapy. (2021) 23:908–17. doi: 10.1016/j.jcyt.2021.06.002 34312069

[36] Kong X Zheng J Liu X Wang W Jiang X Chen J. High TRGV 9 subfamily expression marks an improved overall survival in patients with acute myeloid leukemia. Front Immunol. (2022) 13:823352. doi: 10.3389/fimmu.2022.823352 35222403 PMC8866455

[37] Chen D Guo Y Jiang J Wu P Zhang T Wei Q. γδ T cell exhaustion: opportunities for intervention. J Leukoc Biol. (2022) 112:1669–76. doi: 10.1002/JLB.5MR0722-777R PMC9804355 36000310

[38] Davies D Kamdar S Woolf R Zlatareva I Iannitto ML Morton C. PD-1 defines a distinct, functional, tissue-adapted state in Vδ1+ T cells with implications for cancer immunotherapy. Nat Cancer. (2024) 5(3):420–32. doi: 10.1038/s43018-023-00690-0 PMC10965442 38172341

[39] Song Y Wang B Song R Hao Y Wang D Li Y. T-cell immunoglobulin and ITIM domain contributes to CD8+ T-cell immunosenescence. Aging Cell. (2018) 17:e12716. doi: 10.1111/acel.12716 29349889 PMC5847879

[40] Minnie SA Kuns RD Gartlan KH Zhang P Wilkinson AN Samson L. Myeloma escape after stem cell transplantation is a consequence of T-cell exhaustion and is prevented by TIGIT blockade. Blood. (2018) 132:1675–88. doi: 10.1182/blood-2018-01-825240 30154111

[41] Gournay V Vallet N Peux V Vera K Bordenave J Lambert M. Immune landscape after allo-HSCT: TIGIT- and CD161-expressing CD4 T cells are associated with subsequent leukemia relapse. Blood. (2022) 140:1305–21. doi: 10.1182/blood.2022015522 35820057

[42] Qiu D Liu X Wang W Jiang X Wu X Zheng J. TIGIT axis: novel immune checkpoints in anti-leukemia immunity. Clin Exp Med. (2023) 23:165–74. doi: 10.1007/s10238-022-00817-0 35419661

[43] Jin S Zhang Y Zhou F Chen X Sheng J. TIGIT: A promising target to overcome the barrier of immunotherapy in hematological Malignancies. Front Oncol. (2022) 12:1091782. doi: 10.3389/fonc.2022.1091782 36605439 PMC9807865

[44] Ribot JC Lopes N Silva-Santos B. γδ T cells in tissue physiology and surveillance. Nat Rev Immunol. (2021) 21:221–32. doi: 10.1038/s41577-020-00452-4 33057185

[45] Tough DF Rioja I Modis LK Prinjha RK. Epigenetic regulation of T cell memory: recalling therapeutic implications. Trends Immunol. (2020) 41:29–45. doi: 10.1016/j.it.2019.11.008 31813765

[46] Zhou J Shen X Huang J Hodes RJ Rosenberg SA Robbins PF. Telomere length of transferred lymphocytes correlates with *in vivo* persistence and tumor regression in melanoma patients receiving cell transfer therapy. J Immunol. (2005) 175:7046–52. doi: 10.4049/jimmunol.175.10.7046 PMC1351312 16272366

[47] Rosenberg SA Yang JC Sherry RM Kammula US Hughes MS Phan GQ. Durable complete responses in heavily pretreated patients with metastatic melanoma using T-cell transfer immunotherapy. Clin Cancer Res. (2011) 17:4550–7. doi: 10.1158/1078-0432.CCR-11-0116 PMC3131487 21498393

[48] Yao D Xu L Tan J Zhang Y Lu S Li M. Re-balance of memory T cell subsets in peripheral blood from patients with CML after TKI treatment. Oncotarget. (2017) 8:81852–9. doi: 10.18632/oncotarget.20965 PMC5669853 29137227

[49] Gattinoni L Klebanoff CA Restifo NP. Paths to stemness: building the ultimate antitumour T cell. Nat Rev Cancer. (2012) 12:671–84. doi: 10.1038/nrc3322 PMC6352980 22996603

[50] Ren EH Deng YJ Yuan WH Wu ZL Zhang GZ Xie QQ. An immune-related gene signature for determining ewing sarcoma prognosis based on machine learning. J Cancer Res Clin Oncol. (2021) 147:153–65. doi: 10.1007/s00432-020-03396-3 PMC11802167 32968877

[51] Manieri NA Chiang EY Grogan JL. TIGIT: A key inhibitor of the cancer immunity cycle. Trends Immunol. (2017) 38:20–8. doi: 10.1016/j.it.2016.10.002 27793572

[52] Berger C Jensen MC Lansdorp PM Gough M Elliott C Riddell SR. Adoptive transfer of effector CD8+ T cells derived from central memory cells establishes persistent T cell memory in primates. J Clin Invest. (2008) 118:294–305. doi: 10.1172/JCI32103 18060041 PMC2104476

[53] Kang S Wang L Xu L Wang R Kang Q Gao X. Decitabine enhances targeting of AML cells by NY-ESO-1-specific TCR-T cells and promotes the maintenance of effector function and the memory phenotype. Oncogene. (2022) 41:4696–708. doi: 10.1038/s41388-022-02455-y PMC9568428 36097193

[54] Hargadon KM. Tumor microenvironmental influences on dendritic cell and T cell function: A focus on clinically relevant immunologic and metabolic checkpoints. Clin Transl Med. (2020) 10:374–411. doi: 10.1002/ctm2.37 32508018 PMC7240858

[55] Mazo IB Honczarenko M Leung H Cavanagh LL. Bone marrow is a major reservoir and site of recruitment for central memory CD8+ T cells. Immunity. (2005) 22:259–70. doi: 10.1016/j.immuni.2005.01.008 15723813

