# Supplementary material for: Higher TIGIT+ γδ TCM cells may predict poor prognosis in younger adult patients with non-acute promyelocytic AML
Source: Front Immunol. 2024 Apr 22;15:1321126. doi: 10.3389/fimmu.2024.1321126 (PMC11070478; doi:10.3389/fimmu.2024.1321126)
Supplement: Supplementary file 1 [file Table_1.docx]

Supplement Tables

**Supplement Table 1 |** Comparison of percentages of γδ T cell subsets in patients with the AML-M2 and M5 subtypes.

|  | M2 | M5 | *P* value |
| --- | --- | --- | --- |
| γδ T cells | 7.08 | 5.47 | 0.666 |
| T_N_ γδ | 8.08 | 16.30 | 0.863 |
| T_CM_ γδ | 48.30 | 37.20 | 1.000 |
| T_EM_ γδ | 16.50 | 21.30 | 0.666 |
| T_EMRA_ γδ | 9.69 | 25.20 | 0.546 |
| TIGIT+ γδ | 24.70 | 38.30 | 0.031 |
| TIGIT+ T_CM_ γδ | 20.30 | 31.70 | 1.000 |
| TIGIT+ T_EM_ γδ | 9.83 | 22.10 | 0.863 |
| TIGIT+ T_EMRA_ γδ | 67.70 | 76.70 | 0.931 |

**Supplement Table 2 |** Distribution of TIGIT (as percentages of positive cells) on γδ T cell subsets in PB and BM from patients with AML.

|  | PB | BM | *P* value |
| --- | --- | --- | --- |
| γδ T cells | 7.20 | 9. 44 | 0.002 |
| T_N_ γδ | 8.09 | 14.00 | 0.016 |
| T_CM_ γδ | 34.00 | 25.35 | 0.023 |
| T_EM_ γδ | 18.15 | 18.60 | 0.438 |
| T_EMRA_ γδ | 17.35 | 21.45 | 0.255 |
| TIGIT+ γδ | 33.30 | 60.60 | 0.004 |
| TIGIT+ T_CM_ γδ | 16.65 | 31.20 | 0.007 |
| TIGIT+ T_EM_ γδ | 8.64 | 21.85 | 0.003 |
| TIGIT+ T_EMRA_ γδ | 80.50 | 89.15 | 0.140 |

PB, peripheral blood; BM, peripheral blood.
