# Supplementary figures and images for: Higher TIGIT+ γδ TCM cells may predict poor prognosis in younger adult patients with non-acute promyelocytic AML
Source: Front Immunol. 2024 Apr 22;15:1321126. doi: 10.3389/fimmu.2024.1321126 (PMC11070478; doi:10.3389/fimmu.2024.1321126)

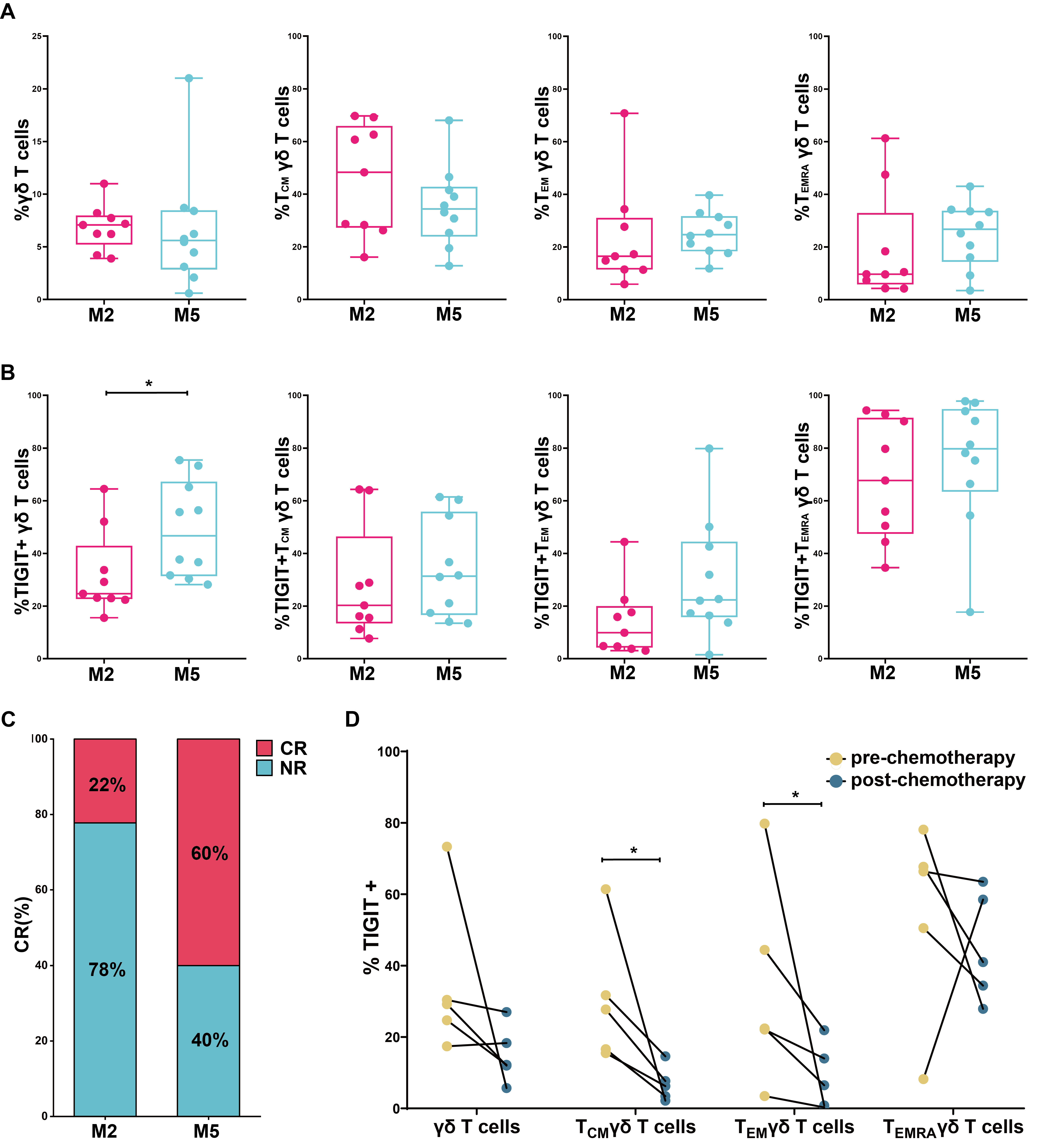

Supplement: Supplementary Figure 1 — Different distribution pattern of TIGIT on γδ T cells between the AML-M2 and AML-M5. (A) The distribution of γδ T cells and their memory subsets in M2 and M5 subtypes. (M2: n = 9, M5: n = 10). (B) Frequency of TIGIT in the TCM, TEM, and TEMRA γδ T-cell populations in M2 and M5 subtypes (M2: n = 9, M5: n = 10). (C) Distribution of CR or NR in M2 and M5 subtypes (M2: n = 9, M5: n = 10). (D) Pairwise comparisons of TIGIT in the γδ T-cell subsets by pre- and postinduction chemotherapy (pre-chemotherapy: n = 5, post-chemotherapy: n = 5). *p < 0.05, **p < 0.01, and ***p < 0.001. [file Image_1.tif]
